# The usefulness of C-reactive protein as a biomarker in predicting neonatal sepsis in a sub-Saharan African region

**DOI:** 10.1186/s13104-020-05033-1

**Published:** 2020-04-01

**Authors:** Gabriel Kambale Bunduki, Yaw Adu-Sarkodie

**Affiliations:** 1 Department of Infectious Diseases, Faculty of Medicine, Université Catholique du Graben, PO. Box 29, Butembo, North-Kivu Democratic Republic of the Congo; 2 Department of Clinical Microbiology, School of Medical Sciences, College of Medicine, Kwame Nkrumah University of Science and Technology, Kumasi, Ghana

**Keywords:** C-reactive protein, Biomarker, Blood culture, Neonatal sepsis, DRC, Sub-Saharan Africa

## Abstract

**Objective:**

The early diagnosis of neonatal sepsis remains a challenge for physicians. The initiation or/and discontinuation of the empirical antibiotic therapy at neonates with sepsis is a dilemma due to the lack of definitive diagnosis and the fear of misdiagnosing a case with its serious outcomes, which can follow up. Therefore, this study aimed to assess the usefulness of C-reactive protein (CRP) as an inflammatory biomarker in the prediction of the neonatal sepsis diagnosis in Butembo, the Democratic Republic of the Congo, in sub-Saharan Africa. Blood culture and quantitative CRP measurements were performed for each neonate. Receiver operating characteristics (ROC) analyses were done in the assessment of CRP accuracy in diagnosing neonatal sepsis.

**Results:**

Of the 228 neonates screened for sepsis, 69 (30.3%) had a positive blood culture. Of the 228 neonates with suspected sepsis, 94 (41.2%) had a positive CRP. Among the 69 cases with positive blood culture, CRP identified 66 cases. The sensitivity, specificity, positive and negative predictive values of CRP were 95.7%, 82.4%, 70.2%, and 97.8%, respectively. The area under the curve (AUC) for the CRP ROC analysis was 0.948. CRP showed its usefulness in the diagnosis of neonatal sepsis.

## Introduction

Blood culture is the gold standard for the diagnosis of neonatal sepsis. However, the results reports are usually available beyond 2 to 3 days [1]. Meanwhile, the early initiation of antibiotic therapy should be done to reduce morbidity and mortality due to sepsis. Due to the lack of the definitive diagnosis and as signs and symptoms of neonatal sepsis are non-specific, the empiric antibiotic therapy may result in treating up to 30 uninfected neonates for a single one who is probably diagnosed to be infected [2].

Other blood investigations that may be done in diagnosing neonatal sepsis include full blood count and acute phase reactants, such as the C-reactive protein (CRP) [3]. However, the concentration of this biomarker (CRP) is time-dependent on the onset of infection. It may raise as much as a thousand fold within 4 to 6 h of an inflammatory process and should be therefore performed 6 to 12 h after the onset of the inflammatory response [3, 4]. CRP levels rapidly decline within an elimination half-life of 19 h upon resolution of the inflammation [5]. This infectious inflammatory biomarker may complement the assessment of clinical signs and risk factors within the diagnosis of neonatal sepsis [6]. The total white blood cells count does not help diagnose neonatal sepsis as it has a low positive predictive value [7]. Several studies are currently assessing laboratory inflammatory biomarkers in the diagnosis of neonatal sepsis. Recent studies among them have suggested the use of CRP biomarker [8, 9] for the precocious diagnosis of neonatal sepsis. It has been shown that no combination of biomarkers performs well in diagnosing sepsis like CRP alone [9]. Current available diagnostic tools are not so useful in the decision to initiate empiric antibiotic therapy in neonates suspected with sepsis but may help in the decision to discontinue antibiotic therapy [10]. CRP has shown to be helpful to decrease antibiotic use [11]. The raised of CRP concentration in septic individuals correlates well with organ failure and increases risk of death [12]. In the absence of methods for detecting the pathogenic bacterial agent, sepsis is diagnosed using clinical signs and increases in CRP concentrations [13]. CRP concentration is not affected by a prior taking of antibiotics, unlike blood culture [14]. In most sub-Saharan African countries like the Democratic Republic of the Congo, a large number of neonates receive antibiotics before their admission at the hospital [15].

The measurement of biomarkers associated with different risk factors represents substantial prediction in diagnosing neonatal sepsis early. Although the use of biomarkers in helping diagnose sepsis has been explored and found to be promising, there is a paucity of data regarding this in sub-Saharan Africa since most of such studies were carried out in developed countries. Therefore, this study aimed to assess the usefulness of CRP as an inflammatory biomarker in the prediction of neonatal sepsis in Butembo, the Democratic Republic of the Congo.

## Main text

### Study design and setting

A cross-sectional study was carried out in three hospitals in Butembo/Eastern DRC within 3 months, from September to November 2018. Hospitals were selected based on their hierarchy in the health system of the DRC, their accessibility, and geographic location regarding the laboratory where samples were processed. Neonates admitted at the concerned health facilities during the study period constituted the study population. Neonates suspected with sepsis, according to the International Paediatric Sepsis Consensus criteria (IPSC) definition (sepsis 2.0), were recruited [16]. In this study, we considered as neonates suspected with sepsis those meeting the IPSC criteria. Neonates meeting the IPSC criteria and with a positive culture were considered as neonates with proven sepsis. Cases, where the parents did not consent to participate in the study, were excluded. All neonates diagnosed with sepsis but who died immediately or upon arrival at the health facility and blood samples were not yet taken were excluded from the study. Neonates with a congenital malformation or dysmorphic features, those diagnosed with malaria parasitaemia, those from HIV-positive mothers, those under antibiotic therapy, and those above 30 days of life were also excluded.

### Sample size and sampling procedure

The sample size estimation was based on previously published prevalence [17] using Fischer’s formula with a maximum error of 5% within a confidence interval (CI) of 95%. Therefore, a total of 228 neonates who met inclusion criteria were screened. Neonates suspected with sepsis were screened by a physician or trained nurse for signs and symptoms of sepsis. One to two millilitres of blood sample were taken before any antibiotics therapy for blood culture and CRP measurement. All the samples were sent to the Central Research Laboratory of the “Université Catholique du Graben” for subsequent processing.

### Processing of the samples

One to one and a half millilitres of blood were aseptically inoculated in a standard bottle for culture. The blood culture and bacterial identification were done as described in by Koneman [18].

Reagents for C-reactive protein were obtained from Robonik^®^, China. These reagents were used for the measurement of CRP using the principle of latex particle-enhanced turbidimetric immunoassay according to the manufacturer’s instructions. Briefly, the principle is summarized as follows. The proteins (CRP) in the sample bind to the specific anti-CRP antibody, which is coated on latex particles and causes agglutination. The degree of the turbidity caused by agglutination is measured optically and is proportional to the concentration of CRP in the sample. The technique consisted of taking 5 μL of the sample (serum, plasma or whole blood); add respectively after 5 min 240 μL of R1 diluent (buffer) and 80 μL of R2 latex reagent, wait 10 min of reaction and then read the optical density using the BioCup GR200 (China) analyser. The cut off of CRP ≥ 6 mg/L was used and considered as positive. This was the optimal value for detecting a neonate with sepsis taking blood culture as the gold standard. The choice of ≥ 6 mg/L CRP threshold was also because in poor setting conditions, there is a qualitative test that can detect a CRP value of ≥ 6 mg/L as positive.

### Data analysis

Study data were captured into a Microsoft Excel 2010 spread sheet-work and exported into the Statistical Package for the Social Sciences (SPSS) version 22 for editing and statistical analyses. Summaries of measures were presented as tables, figures and percentages. Comparisons between categorical data were conducted with Fisher’s exact test or Chi-square. Multivariate logistic regression was used for assessing associations between the CRP level and the independent’s exposure variables. Adjusted Odds ratio (AOR) and their corresponding 95% confidence intervals (CIs) were obtained. Point estimates of statistical significance were indicated with 2-tailed *P*-values < 0.05.

Receiver operating characteristics (ROC) analyses were used by analysing the area under the curve (AUC), specificity, sensitivity, positive predictive value (PPV), negative predictive value (NPV) of the CRP taking the blood culture as the gold standard of neonatal sepsis diagnosis.

### Results

**Fig. 1:**
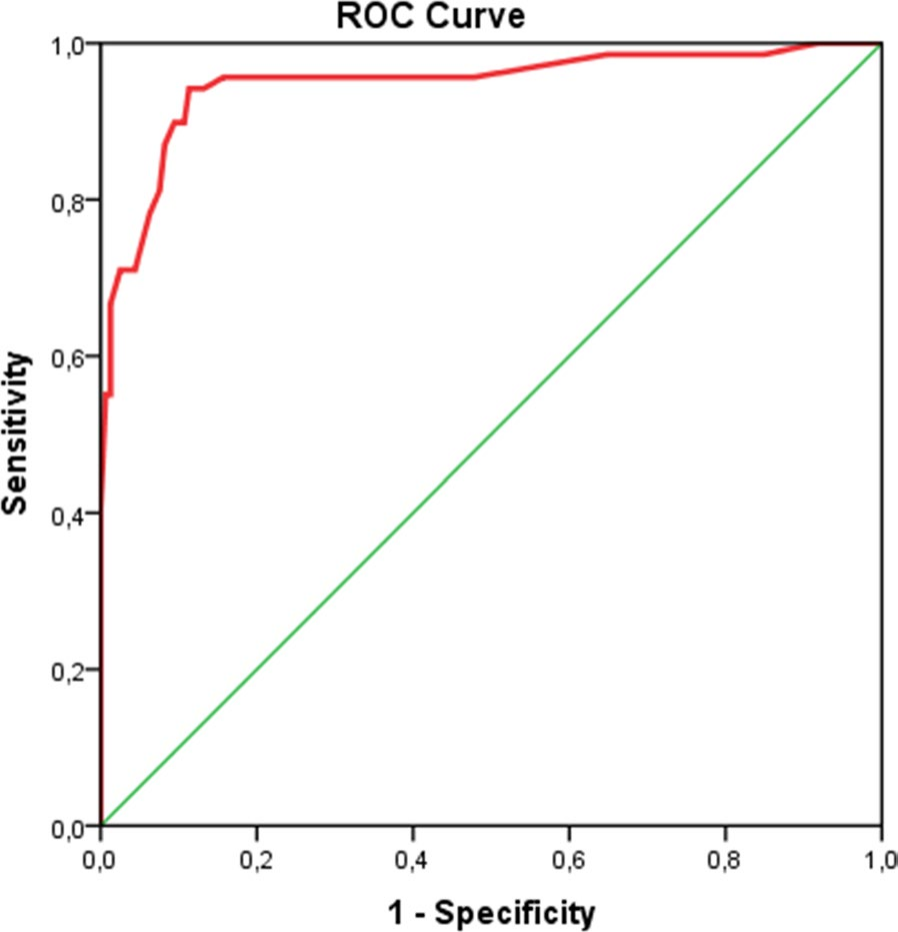
Receiver operating characteristics (ROC) curve of CRP in diagnosing neonates with sepsis (AUC: 0.948, standard error: 0.018; 95% CI 0.913–0.984)

Two hundred and twenty-eight neonates were screened for sepsis. Of the 228 neonates screened, 69 (30.3%) had a positive blood culture, while 159 (69.7%) had a negative blood culture. Of the 228 neonates with suspected sepsis, 94 (41.2%) had a positive CRP, while 134 (58.8%) had a negative CRP. Among the 69 cases with positive blood culture, the CRP identified 66 cases. The sensitivity, specificity, positive, and negative predictive values of the CRP were 95.7%, 82.4%, 70.2%, and 97.8%, respectively (Table 1). The ROC curve of the CRP shows that the area under the curve (AUC) is 0.948 (P < 0.0001, 95% CI 0.913–0.984) (Fig. 1).

**Table 1:** Sensitivity, specificity, positive and negative predictive values of C-reactive protein using blood culture as the gold standard

CRP	Blood culture		Total	P-value	Sensitivity	Specificity	PPV	NPV
	Positive	Negative						
Positive	66	28	94	< 0.000	95.7	82.4	70.2	97.8
Negative	3	131	134					
Total	69	159	228					

*CRP* C-reactive protein, *PPV* positive predictive value, *NPV* negative predictive value

Table 2 shows the performance of the CRP in the neonates with positive blood culture. Of the 69 neonates with positive blood culture, the common bacteria isolated were *Staphylococcus aureus* in 20 (29.0%) cases, *Escherichia coli* in 9 (13.1%) cases, *Streptococcus agalactiae* in 8 (11.6%) cases, coagulase-negative *staphylococci* in 8 (11.6%) cases, *Klebsiella* spp. in 6 (8.7%) cases, and *Pseudomonas aeruginosa* in 6 (8.7%) cases. The CRP was positive in 95.7% of the cases with proven bacteria. Both, early and late onset neonatal sepsis, were taken into account.

**Table 2:** Performance of CRP in the neonate with positive blood culture

Isolated pathogen	CRP results		Total
	Negative	Positive	
*Acinetobacter* spp.	0 (0.0)	3 (100)	3 (4.3)
*Citrobacter* spp.	0 (0.0)	2 (100)	2 (2.9)
*CoNS*	0 (0.0)	8 (100)	8 (11.6)
*Escherichia coli*	1 (11.1)	8 (88.9)	9 (13.1)
*Enterobacter* spp.	0 (0.0)	2 (100)	2 (2.9)
*Enterococcus* spp.	0 (0.0)	3 (100)	3 (4.3)
*Klebsiella* spp.	1 (16.7)	5 (83.3)	6 (8.7)
*Pseudomonas aeruginosa*	0 (0.0)	6 (100)	6 (8.7)
*Streptococcus agalactiae*	0 (0.0)	8 (100)	8 (11.6)
*Staphylococcus aureus*	1 (5.0)	19 (85.0)	20 (29.0)
*Streptococcus pneumoniae*	0 (0.0)	2 (100)	2 (2.9)
Total	3 (4.3)	66 (95.7)	69 (100)

### Discussion

In this study, the CRP identified 66 out of 69 neonates who had a positive blood culture with a sensitivity of 95.7% and a positive predictive value of 70.2%. This implies that a positive CRP will correctly diagnose about 9 of 10 neonates suspected with sepsis, and among them, seven will have a positive blood culture. This is too much higher and can help clinicians to initiate empirical antibiotic therapy for neonates suspected with sepsis. These findings are similar to those of Nuntnarumit et al. in Thailand, who reported a higher sensitivity, specificity, positive and negative predictive values of CRP in diagnosing neonatal sepsis with CRP [19]. In this study, three cases with proven sepsis had a negative CRP. This may be explained by the fact that CRP has the ability to decrease with the rate at which the damaging tissue process resolves [20].

The area under the curve (AUC) of the ROC curve, which was 0.948, confirmed a significant interest for this biomarker. In a recent study, Parlato et al. have found that CRP is the best biomarker which emerged in diagnosing sepsis [9].

From this, a negative CRP can be useful for deciding discontinuation of antibiotic therapy if the clinical features of sepsis are absent. This leads to early discharge from the hospital with a reduced cost of health care, complications of long treatment as well as the family anxiety [14]. Although the CRP shows its usefulness in the diagnosis of neonatal sepsis, it does not substitute the microbiological culture [21]. The sensibility of CRP measurement may vary according to the qualitative or quantitative method used. In this study, the quantitative method was used, and the positive cut off of CRP was ≥ 6 mg/L. The ROC curve plotted showed the best CRP cut off the value of 6 mg/L for giving the best compromise in between the true positive rate (sensitivity) and the false positive rate (1-specificity). This seems to be helpful for sub-Saharan countries since there is a qualitative method used, which consider as positive a CRP level of ≥ 6 mg/L [6]. In these countries, quantitative methods measurements of CRP are not available in all hospitals, and if available, their costs are not affordable for all patients.

The profile of isolates revealed a high rate of *Staphylococcus aureus* followed by *Escherichia coli*, *Streptococcus agalactiae*, Coagulase Negative *Staphylococci* (CoNS), *Klebsiella* spp., *Pseudomonas aeruginosa*, *Enterococcus* spp., *Acinetobacter* spp., *Streptococcus pneumoniae*, *Citrobacter* spp. and *Enterobacter* spp. These isolates are the predominant bacterial causative agents that have been reported in several studies, in early and late onset neonatal sepsis [22–24]. Other studies from developing countries have reported a different bacterial gallery responsible for neonatal sepsis [25, 26]. This difference can be explained by the fact that the bacterial spectrum of neonatal sepsis varies from region to region.

In conclusion, C-reactive protein has shown high performance in early diagnosing cases of neonatal sepsis. Its sensitivity, specificity, positive and negative predictive values were 95.7%, 82.4%, 70.2%, and 97.8%, respectively. Therefore, CRP may be useful in poor resource countries where blood culture is not available or while waiting for blood culture results. It may help deciding of initiation or discontinuation of the empiric antibiotic therapy.

## Limitations

The limitation of this study was that we could not perform the serial CRP assays due to financial constraints. Moreover, CRP results were not correlated to gestational age and birth weight. Hence, further studies using a case–control and heterogeneity approaches need to be done in a large cohort of neonates as the immune response varies depending on gestational age.

## Data Availability

The datasets used and/or analysed during the current study are available from the corresponding author on reasonable request.

## Abbreviations

AOR: Adjusted odds ratio
CI: Confidence interval
CLSI: Clinical Laboratory Standard Institute
CRP: C-reactive protein
CoNS: Coagulase Negative *Staphylococci*
COR: Crude odds ratio
DHS: Demographic Health Survey
DRC: Democratic Republic of the Congo
IPSC: International Paediatric Sepsis Consensus criteria
OR: Odds ratio
ROC: Receiver operating characteristics
AUC: Area under the curve
SD: Standard deviation
PPV: Positive predictive value
NPP: Negative predictive value
DRC: Democratic Republic of the Congo
SPSS: Statistical Package for the Social Sciences
